# Generation of hypothalamic neural stem cell-like cells *in vitro* from human pluripotent stem cells

**DOI:** 10.1016/j.stemcr.2023.02.006

**Published:** 2023-03-23

**Authors:** Tsutomu Miwata, Hidetaka Suga, Yohei Kawaguchi, Mayu Sakakibara, Mayuko Kano, Shiori Taga, Mika Soen, Hajime Ozaki, Tomoyoshi Asano, Hiroo Sasaki, Takashi Miyata, Yoshinori Yasuda, Tomoko Kobayashi, Mariko Sugiyama, Takeshi Onoue, Hiroshi Takagi, Daisuke Hagiwara, Shintaro Iwama, Hiroshi Arima

**Affiliations:** 1 Department of Endocrinology and Diabetes, Nagoya University Graduate School of Medicine, Nagoya, Japan; 2 Division of Metabolism and Endocrinology, Department of Internal Medicine, St. Marianna University School of Medicine, Kanagawa, Japan; 3 Regenerative & Cellular Medicine Kobe Center, Sumitomo Pharma Co., Ltd., Kobe, Japan; 4 Department of Neurosurgery, Graduate School of Medicine, Nagoya University, Nagoya, Japan; 5 Department of Gastroenterology and Metabolism, Nagoya City University Graduate School of Medical Sciences, Nagoya, Japan

**Keywords:** human pluripotent stem cell, hypothalamic neural stem cell, self-renewal, multipotency, neurogenesis, cell-surface antigen, stem cell niche, RAX, hypothalamic organoid, neurosphere

## Abstract

When damaged, restoring the function of the hypothalamus is currently impossible. It is unclear whether neural stem cells exist in the hypothalamus. Studies have reported that adult rodent tanycytes around the third ventricle function as hypothalamic neural stem cell-like cells. However, it is currently impossible to collect periventricular cells from humans. We attempted to generate hypothalamic neural stem cell-like cells from human embryonic stem cells (ESCs). We focused on retina and anterior neural fold homeobox (RAX) because its expression is gradually restricted to tanycytes during the late embryonic stage. We differentiated RAX::VENUS knockin human ESCs (hESCs) into hypothalamic organoids and sorted RAX^+^ cells from mature organoids. The isolated RAX^+^ cells formed neurospheres and exhibited self-renewal and multipotency. Neurogenesis was observed when neurospheres were transplanted into the mouse hypothalamus. We isolated RAX^+^ hypothalamic neural stem cell-like cells from wild-type human ES organoids. This is the first study to differentiate human hypothalamic neural stem cell-like cells from pluripotent stem cells.

## Highlights

• Hypothalamic neural stem cell niche was maintained in organoids induced from hESCs
• Sorted RAX+ cells formed neurospheres and exhibited self-renewal and multipotency
• Transplantation of neurospheres into mice showed neurogenesis
• Sorting by surface antigens and passaging could isolate RAX^+^ cells from WT hESCs

## Graphical abstract

**Figure:**
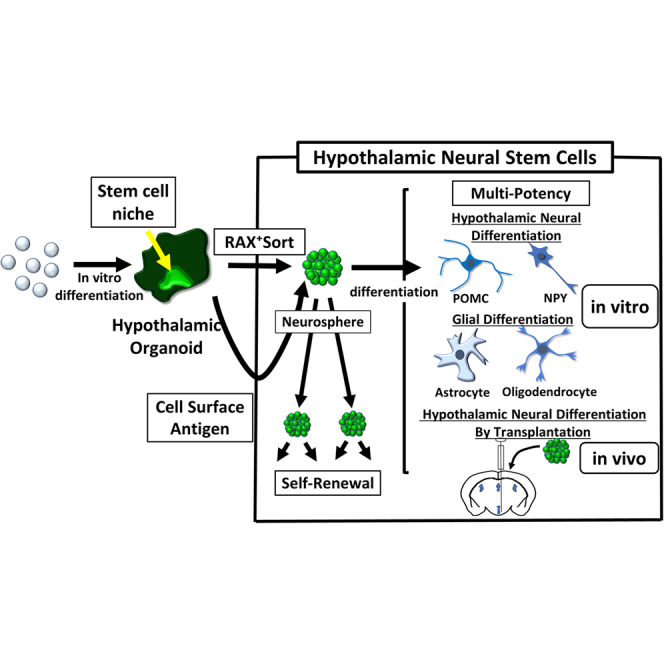

## Teaser

Miwata et al. generated hypothalamic neural stem cells from hESCs, focusing on long-lasting RAX expression, and confirmed their function *in vitro* and *in vivo*. We also established a set of cell-surface antigens to isolate hypothalamic neural stem cells from wild-type hESCs. This technology would be applied to the field of regenerative medicine for patients with hypothalamic disorders.

## Introduction

The hypothalamus is an essential region of the brain that maintains physiological homeostasis. It can be damaged by a number of factors, such as brain tumors, hereditary diseases, and inflammatory diseases (Angulo et al., 2015; Daubenbüchel and Müller, 2015; Rao et al., 2016; Tang et al., 2015). Patients with hypothalamic disorders suffer lifelong symptoms of endocrine disorders, obesity, and associated lifestyle-related diseases such as diabetes (Tauber and Hoybye, 2021). When damaged, it is currently impossible to recover its function. Therefore, treatment for hypothalamic disorders involves only symptomatic therapy, such as hormone replacement therapy, and there is currently no curative treatment.

Recently, tissue generation from pluripotent stem cells has attracted attention as a treatment for such refractory diseases. However, transplantation of mature neuronal cells alone does not result in successful neuronal engraftment (Fricker-Gates et al., 2002). Therefore, trial transplantation of neural progenitor cells or neural stem cells has been applied in clinical practice (Kikuchi et al., 2017; Nori et al., 2011). In contrast, the presence of neural stem cells in the human hypothalamus has been unknown.

Tanycytes are radial glial cell-like ependymal cells found in the ventral hypothalamus of rodents. Recent studies have reported that tanycytes in rodents possess self-renewal and multilineage properties, indicating that they are hypothalamic neural stem cell-like cells (Lee et al., 2012; Robins et al., 2013). Recently, we succeeded in generating tanycyte-like cells from mouse embryonic stem cells (mESCs) and showed that these cells have properties of hypothalamic neural stem cell-like cells (Kano et al., 2019).

However, there are limited reports on whether tanycytes have the same properties in humans (Nogueira et al., 2014; Zhou et al., 2020) because of the difficulty in obtaining human third ventricle periventricular cells. In this study, we induced human hypothalamic neural tissue organoids using human ESCs (hESCs), attempted to fractionate hypothalamic neural stem cell-like cells from the organoids and examined whether these fractionated cells have tissue stem cell properties.

## Results

### Differentiation of hESCs into hypothalamic-pituitary organoids rich in RAX^+^ cells

We focused on the transcription factor retina and anterior neural fold homeobox (RAX) as markers of hypothalamic neural stem cell-like cells. RAX is widely expressed in the hypothalamus and retina of early embryonic mice (embryonic day 7.5 [E7.5]–E13.5). The positive region is gradually limited and is only partially expressed in tanycytes and the retina of late embryonic mice (E17.5) (Pak et al., 2014; Wataya et al., 2008). Based on this property, we hypothesized that cells still expressing RAX in hESC-derived hypothalamic tissues after most of the hypothalamic cells differentiated into neurons and glial cells would correspond to tanycytes. Therefore, we first investigated the induction of RAX-rich hypothalamic tissue in hESCs. We used a RAX::VENUS knockin hESC line to examine the culture method that would result in the highest and longest expression of the RAX::VENUS protein.

**Figure 1:**
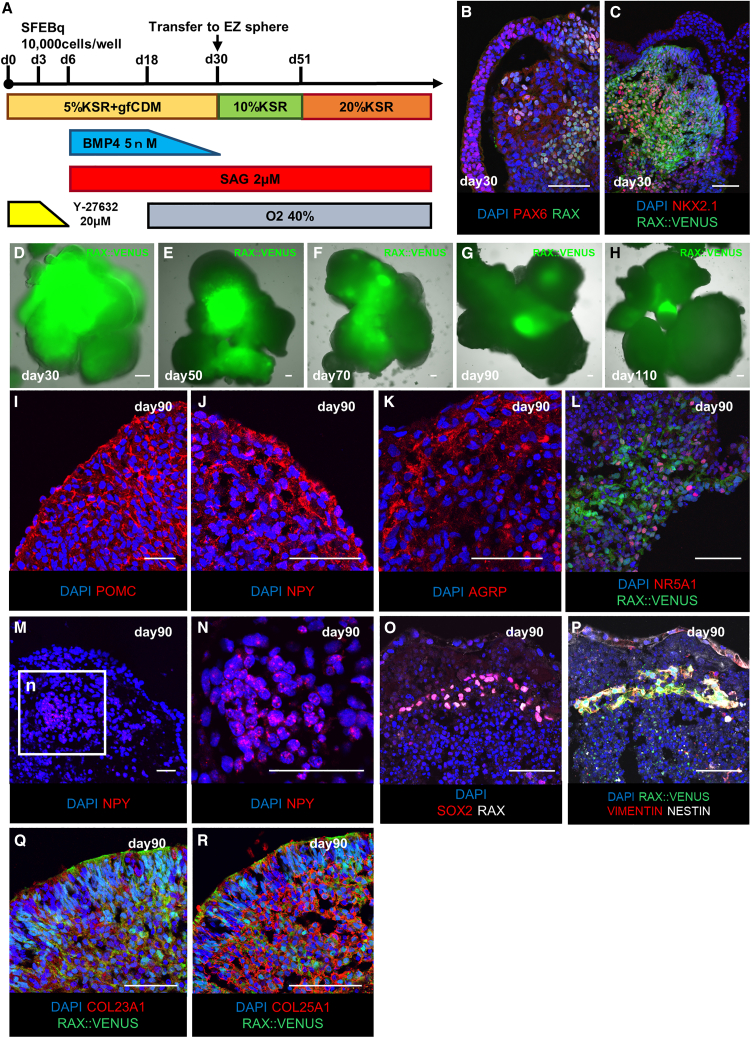
Characteristics of human hypothalamic-pituitary organoids generated with three-dimensional culture (A) Culture protocol for hypothalamic-pituitary organoid induction. (B and C) Immunostaining of hESC aggregates on day 30 for PAX6 (red; B), NKX2.1 (red; C), markers of hypothalamic progenitor cells co-expressed with RAX (green; B), and RAX::VENUS (green; C). For RAX::VENUS, spontaneous luminescence of the VENUS protein was imaged without staining. (D–H) Time series of fluorescence microscopy images of hypothalamic-pituitary organoids; RAX:VENUS^+^ areas (green; D–H) appear to decrease gradually until day 90, and small amounts of positive areas remain after day 90. (I–L) Immunostaining of hESC-derived hypothalamic-pituitary organoids on day 90 for POMC (red; I), NPY (red; J), AGRP (red; K), NR5A1 (red; L), markers of the hypothalamic arcuate nucleus and ventral medial nucleus. (M and N) RNAScope results for NPY (red). (O and P) Immunostaining of the hypothalamic area of the day 90 hypothalamic-pituitary organoid for SOX2 (red; O), VIMENTIN (red; P), NESTIN (white; P), and markers of neural stem cells co-expressed with RAX (white; O). (Q and R) Immunostaining of the hypothalamic area of day-90 hypothalamic-pituitary organoids for COL23A1 (red; Q), COL25A1 (red; R), and markers of tanycytes co-expressed with RAX::VENUS (green; Q and R). Scale bars, 50 μm (B–R).

We used a three-dimensional serum-free culture of embryoid body-like aggregates with quick reaggregation (SFEBq) (Eiraku et al., 2008; Watanabe et al., 2005) to induce differentiation of mouse ESCs into the hypothalamus. Based on our previous differentiation methods (Kasai et al., 2020; Ogawa et al., 2018; Ozone et al., 2016), we compared several induction conditions that could contain hypothalamic tissues differentiated from hESCs/induced pluripotent stem cells (iPSCs). One of the hypothalamic-pituitary organoid induction methods (Ozone et al., 2016) showed the most long-lasting RAX^+^ cells (Figures 1A, S1A, and S1B) compared with the hypothalamic induction method (Ogawa et al., 2018). This result may be due to the basic rule that the hypothalamus interacts with the anterior pituitary to form and mature in embryology (Suga et al., 2011). When the administration period of bone morphogenetic protein 4 (BMP4), which positively affects formation of the oral ectoderm and pituitary anlage, was shortened, RAX expression in the late stage of differentiation decreased from 8.75% ± 0.46% (mean ± SEM, n = 14 independent experiments) to 5.38% ± 0.20% (mean ± SEM, n = 4 independent experiments) (Figures S1B–S1E).

Under this hypothalamic-pituitary organoid differentiation method, the expression of RAX::VENUS protein peaked on day 30, and fluorescence immunostaining showed the expression of paired box genes 6 (PAX6) and nk2 homeobox 1 (NKX2.1), markers of hypothalamic progenitor cells, consistent with RAX^+^ cells (Figures 1B–1D). The hypothalamic precursor cell markers achaete-scute homolog 1 (ASCL1) and neurogenin 2 (NG2) were also expressed, consistent with RAX::VENUS and NKX2.1 (Figures S1F and S1G) (Aslanpour et al., 2020). Then, the expression of RAX::VENUS protein rapidly decreased, and after day 90, there seemed to be no change in RAX::VENUS expression levels (Figures 1D–1H). At that point, ASCL1 and NG2 expression disappeared, suggesting that the hypothalamic precursor cells had matured (Figures S1H and S1I). Immunostaining of the hypothalamic-pituitary organoids on day 90 showed expression of proopiomelanocortin (POMC), neuropeptide Y (NPY), agouti-related peptide (AGRP), and nuclear receptor subfamily 5, group A, member 1 (NR5A1), which are mature neural markers of the arcuate and ventral medial nuclei of the hypothalamus (Figures 1I–1L).

To corroborate this result, we performed several experiments. First, we performed fluorescent immunostaining on adult mice and early hypothalamic organoids (Figures S1J–S1O). The results showed that adult mice and early hypothalamic organoids functioned as positive and negative controls, respectively.

When cultured in three-dimensional floating culture, the cell bodies and axons of neurons are spread out in various directions, making it difficult to obtain a typical image of neurons (Huang et al., 2021). Therefore, we next dispersed the hypothalamic organoids into single cells and cultured them in two-dimensional culture so that the neuropeptides expressed in the hypothalamic organoids would appear more typical (Figures S1P–S1S). The results revealed that the morphology of the hypothalamic organoids was similar to that of previously reported hESC-induced hypothalamic neurons (Wang et al., 2015).

To quantitatively estimate the neuropeptides in the hypothalamic organoids, we performed quantitative polymerase chain reaction (qPCR) and detected the expression of POMC and NPY (Figures S1T–S1V). The reason why AGRP was not detected by qPCR may be that AGRP is expressed in a smaller area in the hypothalamic organoids than POMC or NPY (Figures S1W–S1Y). To confirm that the neuropeptide is expressed in the hypothalamic organoids by methods other than fluorescent immunostaining, we also performed fluorescence *in situ* hybridization (FISH) on NPY and detected NPY in the hypothalamic organoids (Figures 1M and 1N).

These results mean that the aggregates at 90 days were in the final stages of differentiation into neurons and glia; that is, the hypothalamic tissues in the hypothalamic-pituitary organoids in this study matured at 90 days.

Therefore, we hypothesized that the RAX:VENUS^+^ cells expressed even after day 90 were human tanycytes. Immunostaining showed a group of cells in the hypothalamic-pituitary organoids that expressed RAX, sex-determining region Y-box 2 (SOX2), VIMENTIN, and NESTIN in unison, suggesting that they may form hypothalamic stem cell-like cell niches (Figures 1O and 1P). Furthermore, we confirmed that collagen 23 alpha 1 (COL23A1) and collagen 25 alpha 1 (COL25A1), which are known to be expressed in tanycytes, are expressed in hypothalamic organoids consistent with RAX::VENUS, indicating that RAX::VENUS^+^ cells may be the equivalent of tanycytes (Figures 1Q and 1R) (Chen et al., 2017).

### Isolation of RAX::VENUS^+^ cells from hypothalamic-pituitary organoids

**Figure 2:**
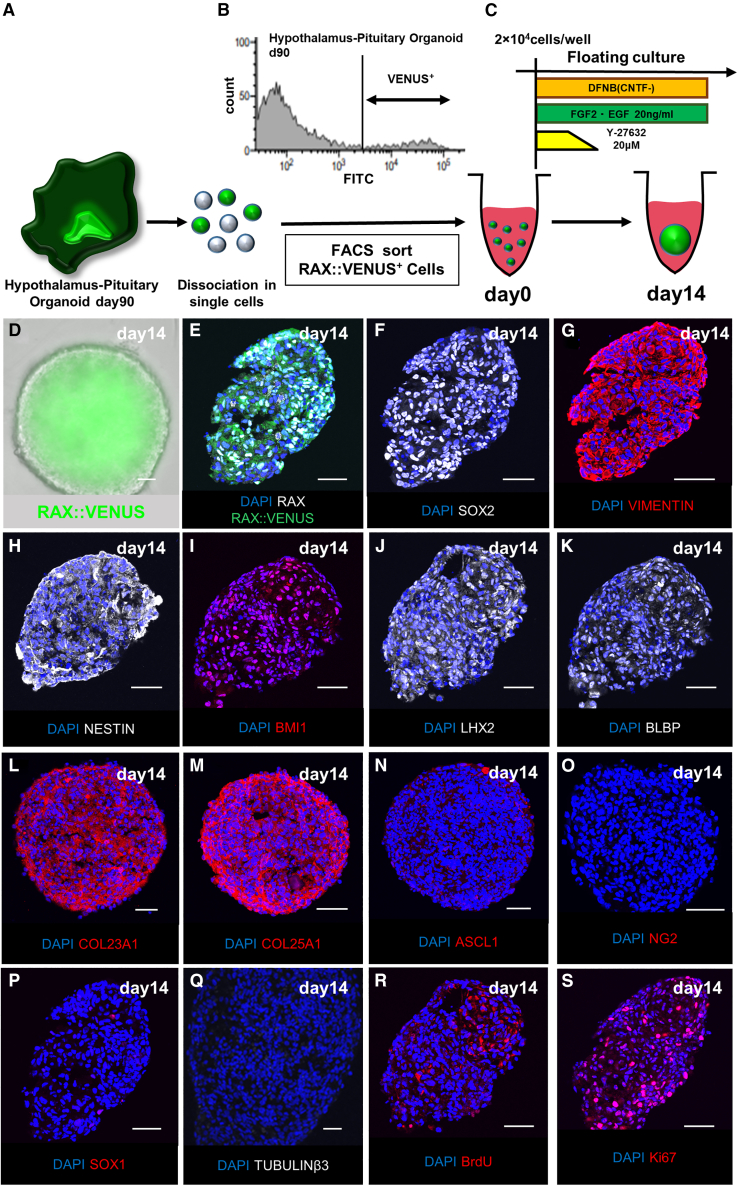
Neurospheres composed of RAX::VENUS^+^ cells isolated from the human hypothalamic-pituitary organoids (A) Schematic of isolation of RAX::VENUS^+^ cells from day 90 hypothalamic-pituitary organoids. (B) Histogram of RAX::VENUS^+^ cells. (C) Culture condition of sorted RAX::VENUS^+^ cells to form neurospheres. (D) Fluorescence microscopy images of the neurosphere composed of RAX::VENUS^+^ cells (green) on day 14 after sorting. (E–S) Immunostaining of the RAX::VENUS (green; E) neurosphere on day 14 for RAX (white; E), SOX2 (white; F), VIMENTIN (red; G), NESTIN (white; H), neural stem cell markers, BMI1 (red; I), a marker expressed for the self-renewal ability of neural stem cells, LHX2 (white; J), BLBP (white; K), COL23A1 (red; L), COL25A1(red; M), expressed in mouse Rax^+^ tanycytes, ASCL1 (red; N), NG2 (red; O), hypothalamic precursor cell markers, SOX1 (red; p), expressed in neural progenitors but not in Rax^+^ tanycytes, TUBULINβ3 (white; Q), mature neuron marker and BrdU (red; R), Ki67 (red; S), cell division markers. Scale bars, 50 μm (D–S).

Next, we attempted to isolate RAX::VENUS^+^ cells from the hESC-derived hypothalamic tissues. Hypothalamic-pituitary organoids were dissociated into single cells on day 90 and sorted for RAX::VENUS^+^ cells using a cell sorter (Figures 2A and 2B). VENUS^+^ cells were defined as those with a fluorescence level of 3 × 10^5^ or greater. This cutoff was determined to contain less than 0.2% of the false-positive cells of undifferentiated hESCs as the VENUS^−^ control (Figure S2A). The ratio of RAX::VENUS^+^ cells sorted on day 90 was 8.75% ± 0.46% (mean ± SEM, n = 14 independent experiments). The percentage of RAX::VENUS^+^ cells on day 90 was significantly decreased compared with that on day 50 (23.3% ± 2.90%, mean ± SEM, n = 3 independent experiments), suggesting that many RAX::VENUS^+^ cells on day 50 differentiated into hypothalamic neural tissue by day 90 (Figure S2B).

In general, neural stem cells form sphere-like aggregates called “neurospheres” when suspended in a medium containing growth factors, such as fibroblast growth factor 2 (FGF2) and epidermal growth factor (EGF) (Reynolds and Weiss, 1992). This characteristic is shared between mouse and human neural stem cells (Nori et al., 2011; Robins et al., 2013). Therefore, we performed a suspension culture of isolated RAX::VENUS^+^ cells in a medium supplemented with FGF2 and EGF to examine whether they formed neurospheres (Figure 2C). We also added a rho-associated protein kinase (ROCK) inhibitor because the single human cells were fragile (Watanabe et al., 2007). First, we used ultra-low-adhesion 24-well plates, which did not aggregate well (Figure S2C). This suggests that the aggregation ability of single human RAX::VENUS^+^ cells is weaker than that of mouse cells. Next, we used 96-well plates, which allowed cells to form aggregates more easily. We observed neurospheres composed entirely of RAX::VENUS^+^ cells (Figure 2D) after 14 days of culture.

Fluorescent immunostaining revealed that the neurospheres composed of RAX::VENUS^+^ cells expressed RAX, SOX2, NESTIN, and VIMENTIN, which are markers expressed in neural stem cells, and B lymphoma Mo-MLV insertion region 1 homolog (BMI1), a marker of the self-renewal ability of neural stem cells (Figures 2E–2I; Goodman and Hajihosseini, 2015; Molofsky et al., 2003). Lim homeobox-2 (LHX2), brain lipid-binding protein (BLBP), COL23A1, and COL25A1, expressed in mouse Rax^+^ tanycytes, were also observed (Figures 2J–2M; Haan et al., 2013). These features suggest that the neurospheres correspond to tanycytes. Interestingly, the neurospheres showed little expression of the hypothalamic precursor cell markers ASCL1, NG2, and SOX1, markers that are expressed in neural progenitor cells but not in Rax^+^ tanycytes, or the mature neuron marker TUBULINβ3 (Figures 2N–2Q and S2D) (Wataya et al., 2008). This suggests that the neurospheres had few impurities, such as hypothalamic precursor cells and neural progenitor cells generated during the differentiation process from ESCs and mature neurons. Expression of bromodeoxyuridine (BrdU) and Ki67, a cell division marker, was also observed. These results indicate that the neurospheres were able to proliferate (Figures 2R and 2S; Lehner et al., 2011; Reif et al., 2006).

### RAX::VENUS^+^ cells have the properties of hypothalamic neural stem cell-like cells *in vitro*

To confirm that tanycytes have properties of hypothalamic neural stem cell-like cells *in vitro*, several studies have reported that neurospheres derived from adult mouse tanycytes can be passaged multiple times and differentiate into hypothalamic neurons and glia (Lee et al., 2012; Robins et al., 2013). Therefore, we examined whether the neurospheres composed of the hESC-derived RAX:VENUS^+^ cells had the same properties as neurospheres derived from adult mouse tanycytes.

**Figure 3:**
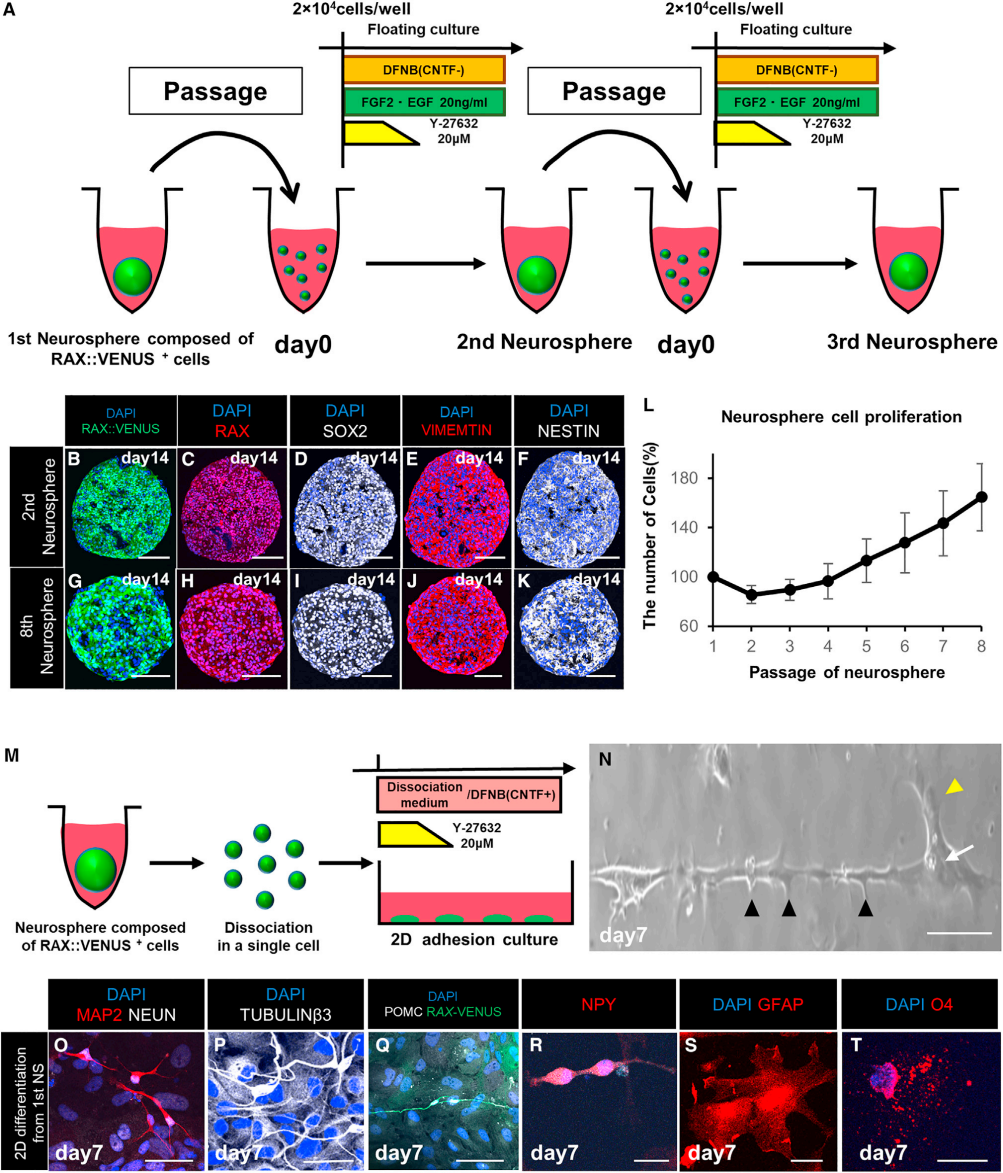
Self-renewal and multipotency of the neurospheres *in vitro* (A) Culture protocol and schematics for passaging the neurospheres. (B–K) Immunostaining of the neurospheres after multiple passages. The neurosphere after one passage (second neurosphere) and the neurosphere after seven passages (eighth neurosphere) expressed RAX (red; C and H), SOX2 (white; D and I), VIMENTIN (red; E and J), NESTIN (white; F and K), neural stem cell markers, consistent with RAX:VENUS (green; B and G). (L) Graph of the proliferation potential of the neurospheres with several passages. The neurospheres were dissociated into single cells at the time of passaging, and the numbers of cells were measured to calculate how much they increased compared with the initial number of cells. Values shown on graphs represent the mean ± SEM; n = 3 independent experiments. (M) Culture protocol to differentiate the neurospheres into neurons and glia. (N) Phase-contrast microscopy image of a neuron differentiated from the neurosphere (first neurosphere) composed of RAX::VENUS^+^ cells. Axons (arrowheads) and dendrites (yellow arrowheads) appeared to extend from the cell body (arrow). (O–T) Immunostaining of neuronal and glial cells differentiated from the neurospheres. Expression of the mature neuron markers MAP2 (red; O), NEUN (white; O), and TUBULINβ3 (white; P); the ventral hypothalamic neuron markers POMC (white; Q) and NPY (red; R); the astrocyte marker GFAP (red; S); and the oligodendrocyte marker O4 (red; T) was observed. Scale bars, 50 μm (B–K, N–T).

First, we dissociated the neurospheres into single cells, added FGF2 and EGF, and suspended them in floating culture in 96-well plates. As a result, new neurospheres were successfully reaggregated; that is, they were successfully passaged (Figure 3A). This passaging could be performed multiple times (more than eight times) while maintaining neural stem cell markers and RAX::VENUS^+^ cells (Figures 3B–3K). We measured the number of cells during passaging and found that the number gradually increased. In other words, RAX::VENUS^+^ cells self-proliferated (Figure 3L). The slow proliferation rate of these cells may reflect the low functional recovery ability of human hypothalamic tissue. These results indicate that the neurospheres composed of RAX:VENUS^+^ cells have self-renewal abilities.

Next, we examined whether the neurospheres composed of RAX::VENUS^+^ cells were multipotent. The neurospheres were dissociated into single cells and attached to PDL-coated cover glasses in a medium supplemented with ciliary neurotrophic factor (CNTF) and ROCK inhibitor (Figure 3M). Neuron-like structures were observed on day 7 (Figure 3N). Immunostaining of these structures showed that they expressed microtubule-associated protein 2 (MAP2), neuronal nuclei (NEUN), and TUBULINβ3; the mature neuron markers POMC and NPY; and ventral hypothalamic arcuate nucleus markers, suggesting that they were differentiated into hypothalamic neurons (Figures 3O–3R). These structures also expressed glial fibrillary acidic protein (GFAP; an astrocyte marker) and O4 (an oligodendrocyte marker), indicating that they had differentiated into two types of glial cells (Figures 3S and 3T). These data show that the neurospheres are multipotent. In addition, we confirmed that the neurospheres had the same multipotency even after passaging (Figures S2E–S2P). In contrast, passaging altered the ratio of differentiation of RAX^+^ cells into hypothalamic neurons and glial cells (Figures S2Q–S2S). The results showed that the percentage of RAX^+^ cells differentiating into mature neurons decreased and that of glial cells increased as the number of passages increased, as reported previously (Okada et al., 2008).

### Transplanted hESC-derived RAX::VENUS^+^ cells are differentiated into hypothalamic neurons *in vivo*

In the experiments above, we have shown that neurospheres composed of RAX::VENUS^+^ cells have the properties of hypothalamic neural stem cell-like cells *in vitro*. Next, we examined whether the neurospheres had the same properties *in vivo*.

**Figure 4:**
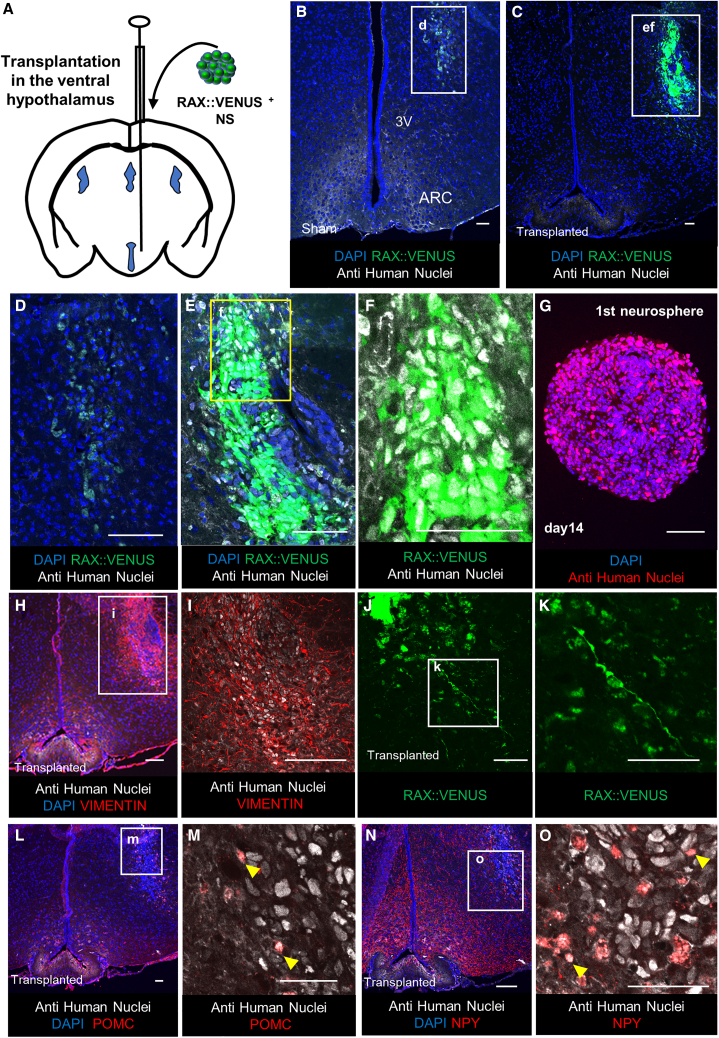
Multipotency of neurosphere *in vivo* transplantation (A) Schematic of the transplantation of hESC-derived RAX::VENUS^+^ neurospheres into the SCID mouse hypothalamus. (B–F and H–O) Immunostaining of a coronal section of a 9-week-old treated SCID mouse brain. (B and D) Ventral hypothalamus of sham-group mice. Very weak expression of the anti-human nucleus antibody (white) and RAX::VENUS (green), not consistent with DAPI, was observed, suggesting that they were non-specific. (C, E, and F) Ventral hypothalamus from the neurosphere transplantation group. The transplanted RAX::VENUS^+^ (green; C, E, and F) and anti-human nuclei antibody^+^ (white; C, E, and F) cell mass was observed. (G) Anti-human nucleus staining (red) of the neurosphere composed of RAX::VENUS^+^ cells. (H and I) Ventral hypothalamus of the neurosphere transplantation group stained for VIMENTIN (red; G and H), which appears to extend axons from the anti-human nuclei antibody^+^ cell mass into the mouse brain. (J and K) Ventral hypothalamus of the neurosphere transplantation group. Bipolar radial glial cell-like RAX::VENUS^+^ cells (K; green) are seen in the area away from the transplanted neurospheres (J; top left). (L–O) Ventral hypothalamus of the neurosphere transplantation group. Shown is immunostaining with ventral hypothalamic markers. Expression of POMC (red; L and M) and NPY (red; N and O), consistent with anti-human nucleus antibody (white; L–O), was observed in a part of the engrafted neurospheres. Scale bars, 50 μm (B–O).

Several studies have shown that tanycytes are capable of neurogenesis (differentiation into hypothalamic neurons) in the adult mouse brain (McNay et al., 2012). Therefore, we transplanted neurospheres composed of RAX::VENUS^+^ cells into the ventral hypothalamus of severe combined immunodeficiency (SCID) mice and tested whether the neurospheres would show neurogenesis in the SCID mouse brain (Figure 4A).

We implanted one neurosphere (i.e., 20,000 RAX^+^ cells) into the mouse ventral hypothalamus. One week after neurosphere transplantation, the neurospheres transplantation group, compared with the sham group, contained cell clusters that expressed RAX:VENUS and anti-human nuclear antibodies, which were also expressed in the hESC-derived neurospheres (Figures 4B–4G). The anti-human nuclear antibodies were expressed in the mouse hypothalamic arcuate nucleus in sham groups; however, the nuclei were not stained, suggesting non-specific expression (Figure S3A). Consistent with these anti-human nuclear antibody^+^ cells, we observed VIMENTIN^+^ cells extending into the mouse brain compared with those in the sham group (Figures 4H, 4I, S3B, and S3C). These results suggest that hESC-derived neurospheres were engrafted into the mouse brain.

Some RAX::VENUS^+^ cells derived from hESCs showed a radial glial cell-like morphology in the transplanted SCID mouse brain, which is the typical appearance of neural stem cells in the mouse brain *in vivo* (Figures 4J and 4K). Some of the anti-human nuclear antibody^+^ cells transplanted into the mouse brain expressed POMC and NPY, which are arcuate nucleus markers of the ventral hypothalamus, compared with those in the sham group, which showed only scarring changes (Figures 4L–4O) (Figures S3D–S3G). These results suggested that these cells differentiate into hypothalamic neurons in the mouse brain. We transplanted the neurosphere into four mice and confirmed its viability in two mice. No tumorigenesis occurred.

These data suggest that the neurospheres composed of RAX::VENUS^+^ cells have the potential for neurogenesis (that is, differentiation into hypothalamic neurons) and have the properties of hypothalamic neural stem cell-like cells *in vivo*.

### Establishment of a method isolating human hypothalamic neural stem cell-like cells using cell-surface antigens

To date, we have used knockedin VENUS as a marker for cell sorting. To make experiments using hypothalamic neural stem cell-like cells widely and conveniently available, we would like to enable their isolation from non-transgenic cells. Therefore, we attempted to isolate cells corresponding to RAX^+^ cells using cell-surface antigens that do not require gene transfer.

**Figure 5:**
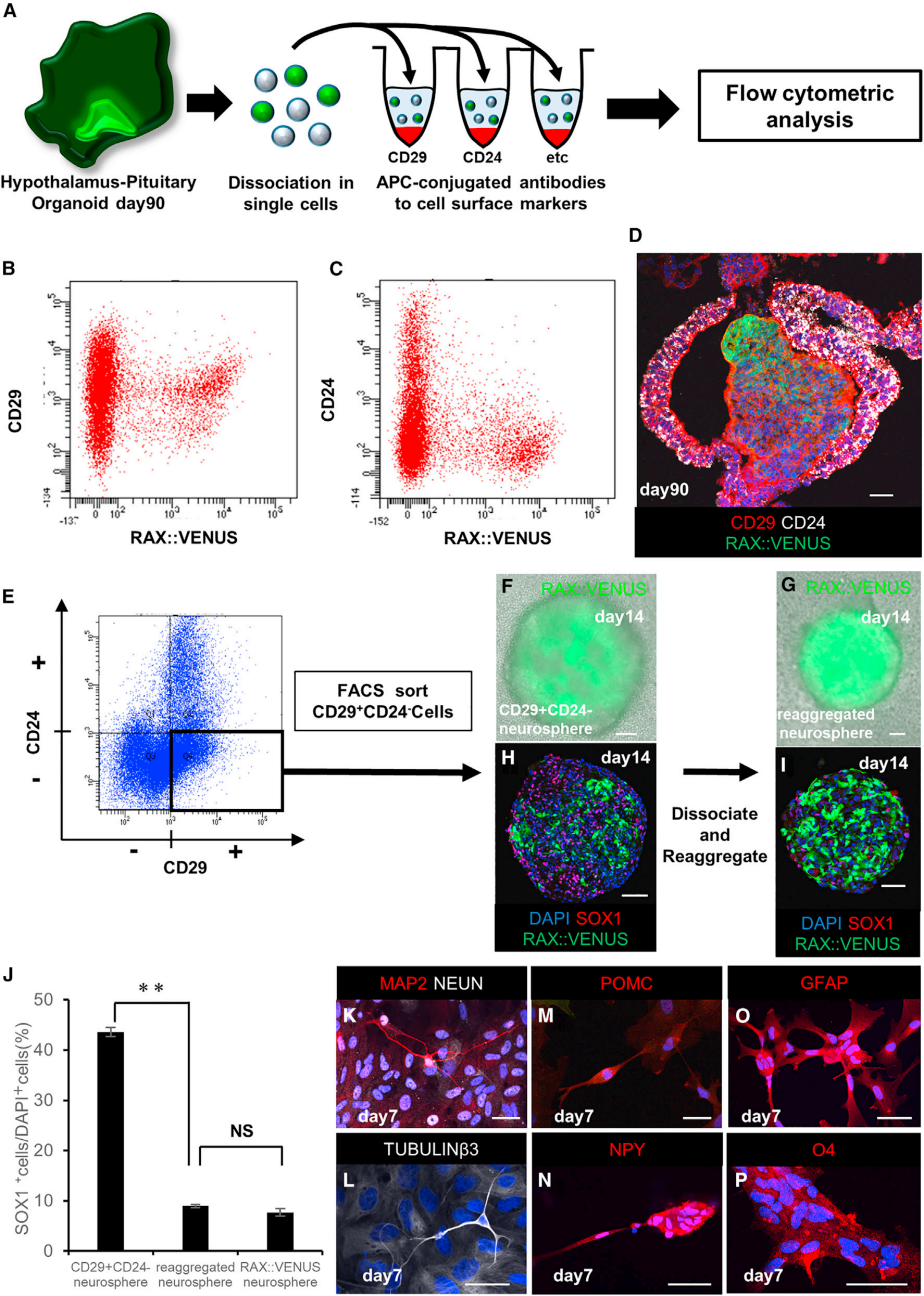
Isolation of human hypothalamic neural stem cell-like cells using cell-surface antigens (A) Schematic of screening using the cell-surface antigen screening kit. (B and C) Analysis of CD29 (B) and CD24 (C) fluorescence in the cells of human hypothalamic-pituitary organoids, including RAX::VENUS^+^ cells. (D) Immunostaining of human hypothalamic-pituitary organoids containing RAX::VENUS^+^ cells (green) on day 90, stained for CD29 (red) and CD24 (white). (E) Fluorescence analysis of CD29 and CD24 of human hypothalamic-pituitary organoids. The CD29^+^ and CD24^−^ fraction (CD29, fluorescence level 1 × 10^5^ or higher; CD24, fluorescence level 1 × 10^5^ or lower) were sorted. RAX::VENUS^+^ cells were contained at 35.4% in the CD29^+^ and CD24^−^ fraction. (F) Neurosphere formed by floating culture of CD29^+^ and CD24^−^ cells for 14 days (CD29^+^ and CD24^−^ neurosphere). (G) Newly formed neurosphere after one passage from the CD29^+^ and CD24^−^ neurosphere. The CD29^+^ and CD24^−^ neurospheres on day 14 were dissociated into single cells and reaggregated with floating culture for 14 days (reaggregated neurosphere). (H and I) Immunostaining of the CD29^+^ and CD24^−^ neurosphere and the reaggregated neurosphere for SOX1 (red; H and I). Passaging decreased the percentage of SOX1^+^ cells and increased the percentage of RAX::VENUS^+^ cells. (J) Rates of SOX1^+^ cells in each neurosphere. Values shown on the graphs represent the means ± SEM, n = 3 independent experiments. ^∗∗^p < 0.01. (K–P) Immunostaining of neurons and glia differentiated from the CD29 and CD24^−^ neurospheres. Expression of the mature neuron markers MAP2 (red; K), NEUN (white; K), and TUBULINβ3 (white; L); the ventral hypothalamic neuron markers POMC (red; M) and NPY (red; N); the astrocyte marker GFAP (red; O); and the oligodendrocyte marker O4 (red; P) was observed. Scale bars, 50 μm (D, F–I and K–P).

To identify the cell surface antigens corresponding to RAX::VENUS^+^ cells, we used a cell surface antigen screening kit that can search for 378 cell surface antigens (Figure 5A). We found that CD29 (integrin β1) expressed in human neural stem cells efficiently sorted RAX::VENUS^+^ cells (Figure 5B; Hall et al., 2006; Pruszak et al., 2007). However, there were also many RAX::VENUS^−^ cells in the CD29^+^ fraction, suggesting the presence of impurities, such as mature neurons and glia.

To remove impurities, we focused on CD24 (Figure 5C). Several studies have reported that CD24^+^ cells are expressed in mature neurons, glia, and the oral ectoderm but not in neural stem cells (Nieoullon et al., 2005; Papagerakis et al., 2014; Pruszak et al., 2007). Therefore, we hypothesized that separating CD29^+^ and CD24^−^ cells could efficiently sort RAX::VENUS^+^ cells. Fluorescent immunostaining of hESC-derived hypothalamic organoids for CD29 and CD24 showed that CD29^+^ and CD24^−^ cells appeared to express RAX::VENUS^+^ cells (Figure 5D). Therefore, we isolated CD29^+^ and CD24^−^ cells and cultured them in suspension culture in FGF2-and EGF-supplemented medium (Figure 5E). They successfully formed neurospheres (CD29^+^ and CD24^−^ neurospheres) but appeared to be contaminated with impurities because the amount of RAX:VENUS^+^ cells was only 35.4% (Figure 5F) by fluorescence microscopy. Therefore, CD29^+^ and CD24^−^ neurospheres were dissociated into single cells and reaggregated in suspension culture; that is, another passaging operation was performed. As a result, neurospheres consisting of highly purified RAX::VENUS^+^ cells (reaggregated neurosphere; 83.6% ± 4.67% (mean ± SEM, n = 4 independent experiments)) were formed, and impurities in the neurospheres appeared to be removed (Figure 5G).

To confirm whether there were any changes in the properties of the neurospheres before and after reaggregation, we compared them using fluorescence immunostaining. There was no expression of the undifferentiated marker NANOG or octamer-binding transcription factor 3/4 (OCT3/4). This indicated no contamination with undifferentiated hESCs (Figures S4A and S4B) (Ivanova et al., 2006). In addition, expression of the neural stem cell markers SOX2, BMI1, NESTIN, and VIMENTIN was observed, indicating that the cells had properties similar to those of neural stem cells (Figures S4C–S4J). Staining for SOX1 expressed in neural progenitor cells showed that SOX1^+^ cells were consistent with RAX::VENUS^−^ cells (Figures 5H and 5I). The percentage of SOX1 cells in neurospheres revealed that the reaggregated neurospheres (9.00% ± 0.33%, mean ± SEM, n = 3 independent experiments) showed a superior reduction in SOX1^+^ cells compared with the CD29^+^ and CD24^−^ neurospheres (43.6% ± 0.90%, mean ± SEM, n = 3 independent experiments). The reaggregated neurospheres contained almost the same number of SOX1^+^ cells as the neurospheres composed of RAX:VENUS^+^ cells (7.69% ± 0.74%, mean ± SEM, n = 3 independent experiments) (Figure 5J). The reason why SOX1^+^ cells decrease with passaging may be that they are neural progenitors with weaker self-renewal capacity (Galiakberova and Dashinimaev, 2020). In fact, when CD29^+^ CD24^−^ RAX:VENUS cells were isolated and cultured in suspension, neurospheres composed of SOX1^+^ cells were formed but could not be passaged, supporting this hypothesis (Figures S4K–S4N). Therefore, we successfully isolated RAX::VENUS cells from hESC-derived hypothalamic organoids using only cell surface antigens. They showed reaggregation upon passaging, revealing that the cells could self-renew.

Next, to confirm whether the isolated CD29^+^ and CD24^−^ cells had the same multipotency as RAX::VENUS^+^ cells, we dissociated the neurospheres composed of CD29^+^ and CD24^−^ cells into single cells and attached them to PDL-coated cover glasses in CNTF medium with a ROCK inhibitor. Fluorescence immunostaining on day 7 showed that the CD29^+^ and CD24^−^ cells differentiated into hypothalamic neurons and glia, similar to RAX::VENUS^+^ cells, indicating that the neurospheres composed of CD29^+^ and CD24^−^ cells had multipotency (Figures 5K–5P).

## Discussion

We succeeded in generating hypothalamic neural stem cell-like cells from human pluripotent stem cells *in vitro* using RAX as a marker with reference to rodent tanycytes. First, we found a group of cells that were persistently RAX^+^ for a long culture time in hypothalamic-pituitary organoids differentiated from hESCs. The RAX^+^ cells co-expressed neural stem cell markers. Second, we isolated RAX^+^ cells from RAX:VENUS knockin hESC-derived organoids and showed that these cells could differentiate into hypothalamic neurons and glia; that is, multipotency *in vitro*. Third, we showed that human RAX::VENUS^+^ cells can form neurospheres and can be passaged, keeping the expression of neural stem cell markers; that is, they can self-renew. Multipotency was maintained in the neurospheres after multiple passages. Fourth, we transplanted human RAX::VENUS^+^ cells into the mouse hypothalamus and showed that these cells were capable of differentiating into hypothalamic neurons and glia, indicating multipotency *in vivo*. Finally, by analyzing the surface markers, we separated hypothalamic neural stem cell-like cells from wild-type hESCs.

*In vitro* generation of human hypothalamic neural stem cell-like cells using this technology will enable future study of human hypothalamic stem cells. The existence and function of hypothalamic neural stem cell-like cells in human brain could be investigated. This technology will enhance the regenerative capacity of human hypothalamic tissues. For example, in the present study, we transplanted human pluripotent stem cell-derived hypothalamic neural stem cell-like cells into the ventral hypothalamus of mice and confirmed their viability and neurogenesis; that is, their differentiation into hypothalamic neurons. It will be possible to evaluate the function of the transplanted cells using specific knockout animals of hypothalamic neurons or disease models. Furthermore, it has been reported that mouse hypothalamic neural stem cell-like cells are involved in not only regeneration but also aging and metabolic functions based on epigenetic factors (Lee et al., 2012; Xiao et al., 2020; Zhang et al., 2017). Thus, it is possible to study the effects of transplantation on aging in aged animal models. The effects on metabolism can also be explored using human hypothalamic neural stem cell-like cells.

In addition, it would be possible to study the mechanisms underlying the maintenance of hypothalamic neural stem cell-like cells. In fact, FGF2, which is expressed in periventricular cells near the hypothalamic ventromedial nucleus and arcuate nucleus of rodents, was expressed inside the hypothalamic neural stem cell-like cell niche in the hESC-derived organoids, suggesting the existence of a mechanism for long-term maintenance of hypothalamic neural stem cell-like cells in this region (Figures S5A and S5B; Niwa et al., 2016). The hypothalamic neural stem cell-like cell niche that could form neurospheres was sterically maintained for at least 200 days in the cell mass (Figures S5C–S5E). This culture system can be used to elucidate the mechanism of stem cell niche maintenance by spatial transcriptome analysis. In the future, we would like to find ways to increase the number of hypothalamic neural stem cell-like cells and enhance their function.

We currently identified surface markers for isolating hypothalamic neural stem cell-like cells, making this technology widely available. Using surface markers, it has become possible to generate hypothalamic neural stem cell-like cells from a variety of hESC/iPSC lines, which is expected to be a fundamental technology for hypothalamic research in a wide range of fields, such as regenerative medicine, embryology, disease pathology, and aging.

In conclusion, we generated human hypothalamic neural stem cell-like cells *in vitro* using hESCs and demonstrated that they function *in vitro* and *in vivo*.

## Experimental procedures

### Resource availability

#### Corresponding author

Further information and requests for resources and reagents should be directed to and will be fulfilled by the corresponding author, Hidetaka Suga (sugahide@med.nagoya-u.ac.jp).

#### Materials availability

All unique/stable reagents generated in this study are available from the lead contact without restriction.

### qPCR

The procedure is described in the supplemental information.

### RNAScope

The RNAscope Multiplex Fluorescent Reagent Kit v.2 (catalog number 323100) was used on fixed hypothalamic-pituitary organoids. RNA probe mixtures were applied for 2 h at 40°C: RNAscope 3-plex Negative Control Probe (catalog number 320871), RNAscope 3-plex Positive Control Probe-Hu (catalog number 320861), and NPY (catalog number 416671). Amplification and staining steps were performed as described by the manufacturer. Opal 570 reagent (FP1488001KT, red, 1:1,500) was used.

### Cell sorting

RAX::VENUS^+^ cells were sorted using a FACS Melody (BD Biosciences). Data were analyzed using the FACS Diva software (BD Biosciences).

For cell sorting, hypothalamic-pituitary organoids were dissociated into single cells using neuron dissociation solution S (297-78101, Wako Pure Chemicals Industries, Osaka, Japan), collagenase type Ⅰ (037-17603, Wako), and 0.25% trypsin-EDTA (25200056, Gibco). The cell suspension was filtered through 5-mL round-bottom tubes with a cell strainer cap (38030, Falcon, Corning, Corning, NY, USA) before loading.

To avoid cross-contamination, RAX::VENUS^+^ and RAX::VENUS^−^ cells were gated using scatterplots of the undifferentiated hESC population. The sorted cells were collected in sorting buffer (DMEM/F-12 with 1 mM EDTA and 1% fetal bovine serum [FBS]) containing 20 μM Y-27632 and stored at 4°C until plating.

CD29^+^ and CD24^−^ cells were sorted using a FACS Aria (BD Biosciences). Data were analyzed using the FACS Diva software (BD Biosciences). For cell sorting, aggregates were dissociated into single cells using TrypLE (12605-010, Gibco) instead of trypsin-EDTA because trypsin-EDTA strips away the cell-surface antigen (Tsuji et al., 2017). CD29^+^ and CD24^−^ cells were sorted based on the fluorescence intensity that resulted in the highest percentage of RAX::VENUS^+^ cells (sorted RAX-VENUS^+^ cells/sorted all cells). As a result, the most appropriate fluorescence intensity was 1 × 10⁵ or higher for CD29 and 1 × 10⁵ or lower for CD24.

### Neurosphere formation and maintenance

Sorted RAX::VENUS single cells were resuspended in DMEM/F12 supplemented with glucose, N2, and B27 (DFNB) medium, supplemented with 20 ng/mL recombinant human FGF2 (Wako), 20 ng/mL recombinant human EGF (PeproTech), and 20 μM Y-27632. The DFNB medium comprised DMEM/F-12 (D8900, Sigma-Aldrich) containing 3.85 g/L glucose (07-0680-5, Sigma-Aldrich), 1.2 g/L sodium hydrogen carbonate (28-1850-5, Sigma-Aldrich), and 50 U/mL (for penicillin) penicillin/streptomycin (15140-122; Gibco, Waltham, MA, USA) supplemented with 1% N2 (17502-048, Gibco) and 2% B27 (12587-010, Gibco). The cells were then seeded in low-cell-adhesion 96-well plates in V-bottomed conical wells. The cell density was adjusted to 20,000 cells per 200 μL of DFNB medium per well. The cells were incubated at 37°C in a 40% O_2_ and 5% CO_2_ incubator. Half-medium changes were performed every 3 days. The neurospheres were passaged every 14 days. For passaging, the neurospheres were dissociated into single cells using neuron dissociation solution S and seeded according to the original conditions. At the time of passage, the number of cells was determined using a cell counter (OC-C-S02, FPI). To cryopreserve RAX::VENUS^+^ cells, the neurospheres were dissociated into single cells and preserved by vitrification. The same experimental procedure was applied to sorted CD29^+^ and CD24^−^  single cells.

### Neurosphere differentiation

For differentiation, the neurospheres were collected in a 15-mL centrifuge tube and dissociated into single cells using neuron dissociation solution S. The cells were seeded at 100,000 cells per 1 mL in dissociation medium on Poly-D-Lysine (PDL)-coated glass coverslips in 24-well plates and incubated at 37°C in a 5% CO_2_ incubator. The dissociation medium contained DFNB, 10% FBS (SFBM30-2537, Equitech-Bio), BDNF (028-16451, Wako), NT-3 (146-09231, Wako), LM22A-4 (SML0848, Sigma), and 10 μM Y-27632. On day 1, the medium was changed with DFNB plus 10 ng/mL CNTF (257-NT, R&D Systems, Minneapolis, MN, USA) and then every other day. On days 4–7, the cells were fixed for immunostaining.

### Immunohistochemistry

The procedure is described in the supplemental information.

### Transplantation of hESC-derived hypothalamic neural stem cell-like cells

All animal experiments were performed in accordance with our institutional guidelines (Nagoya University ES-0001) for animal studies.

Eight-week-old SCID male mice were anesthetized with 1%–2% isoflurane (Wako) using an animal anesthetization device (MA-AT210D, Muromachi Kikai, Tokyo, Japan). Neurospheres were injected into the ventral hypothalamus via a 23G needle of a 2-μL Hamilton syringe (7102KH), using an ultra-precise stereotactic apparatus (David Kopf Instruments) and coordinates of 1.7 mm posterior to the bregma, 5.4 mm below the surface of the skull, and 0.25 mm lateral to the midline of the brain.

### Cell-surface antigen screening kit experiment

Hypothalamic-pituitary organoids were dissociated into single cells and stained using MACS Marker Screen, human, version 02 (130-127-043,Miltenyi Biotec), which was designed to contain 378 APC-conjugated monoclonal antibodies specific to cell-surface markers and six isotype control antibodies arrayed onto four 96-well plates. For flow cytometry analysis, single-cell suspensions were dispensed into low-cell-adhesion V-bottom 96-well plates at 250,000 cells per well in PEB buffer, which comprised PBS, 0.5% BSA, and 2 mM EDTA. Flow cytometry analysis was performed using a FACS Canto2 (BD Biosciences). Data were analyzed using the FACS Diva software (BD Biosciences).

### Cell counting method for SOX1^+^ cells

The procedure is described in the supplemental information.

### Statistical analyses

Data are expressed as the mean ± SEM. Comparisons between two groups were performed by Student’s t test. Comparisons between multiple groups were performed by one-way ANOVA with post-hoc Bonferroni’s method. n refers to the number of independent experiments. P values of < 0.05 (^∗^) < 0.01 (^∗∗^) were considered statistically significant.

## Author contributions

T.M., H.S., and H.A. designed the study and wrote the manuscript. T.M., Y.K., M. Sakakibara, M. Soen, and H.S. performed the experiments with technical help and advice from M.K., S.T., H.O., T.A., H.S., T.M., Y.Y., T.K., M. Sugiyama, T.O., H.T., D.H., and S.I.

## Data Availability

• The data supporting the results of this study are available within the main paper and supplemental information.

## Supplemental information

Document S1. Figures S1–S5 and Supplemental experimental procedures

Document S2. Article plus supplemental information
