# Supplementary material for: Generation of hypothalamic neural stem cell-like cells in vitro from human pluripotent stem cells
Source: Stem Cell Reports. 2023 Mar 23;18(4):869–83. doi: 10.1016/j.stemcr.2023.02.006 (PMC10147555; doi:10.1016/j.stemcr.2023.02.006)
Supplement: Document S1. Figures S1–S5 and Supplemental experimental procedures [file mmc1.pdf]

Stem Cell Reports, Volume *18*

Supplemental Information

Generation of hypothalamic neural stem cell-like cells *in vitro* from human pluripotent stem cells

Tsutomu Miwata, Hidetaka Suga, Yohei Kawaguchi, Mayu Sakakibara, Mayuko Kano, Shiori Taga, Mika Soen, Hajime Ozaki, Tomoyoshi Asano, Hiroo Sasaki, Takashi Miyata, Yoshinori Yasuda, Tomoko Kobayashi, Mariko Sugiyama, Takeshi Onoue, Hiroshi Takagi, Daisuke Hagiwara, Shintaro Iwama, and Hiroshi Arima

Figure1S

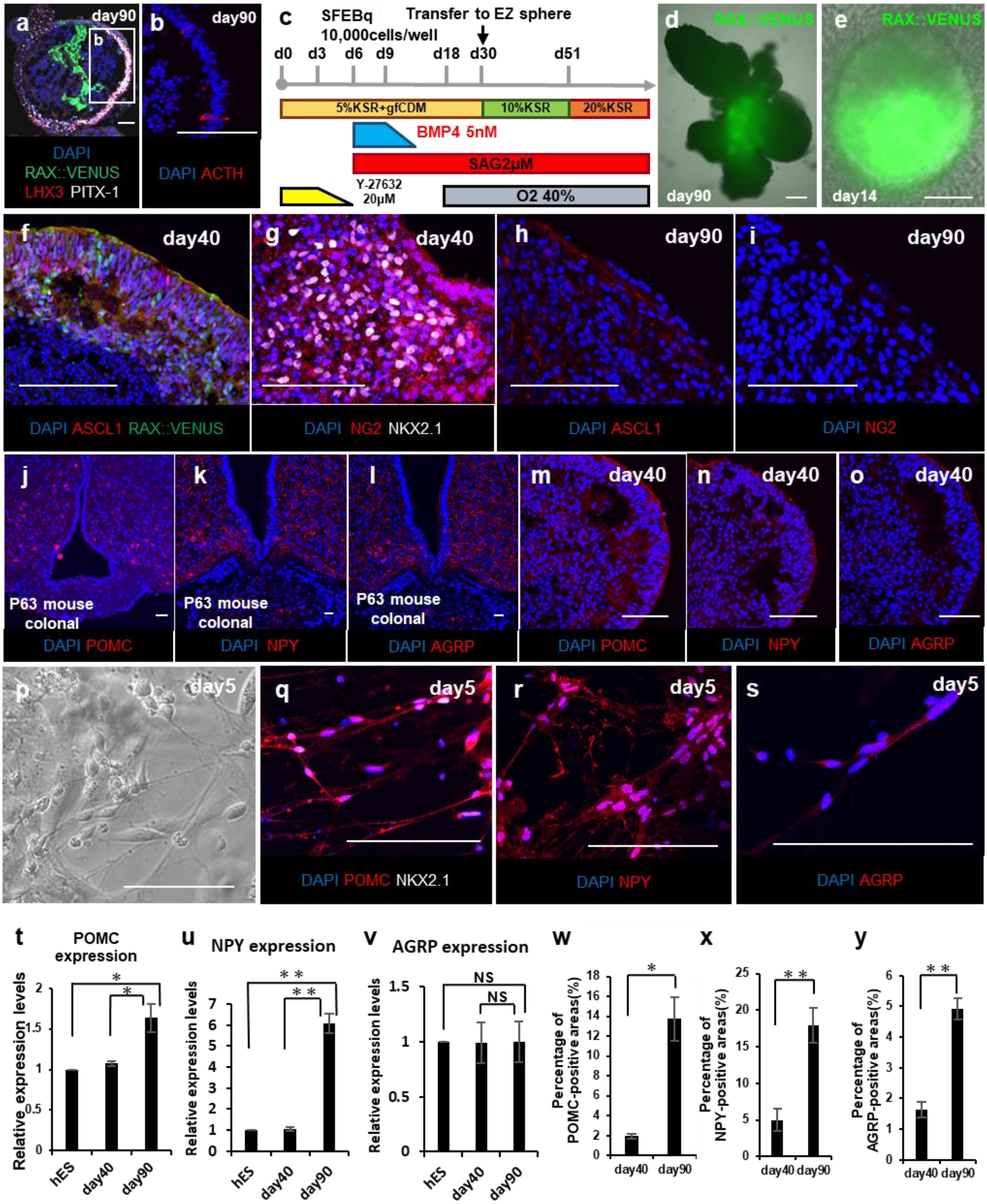

a day90 b
DAPI RAX::VENUS LHX3 PITX-1
b day90
DAPI ACTH
c SFEBq 10,000cells/well Transfer to EZ sphere
d0 d3 d6 d9 d18 d30 d51
5%KSR+gfCDM 10%KSR 20%KSR
BMP4 5nM
SAG2μM
Y-27632 20μM
O2 40%
d RAX::VENUS day90
e RAX::VENUS day14
f day40
DAPI ASCL1 RAX::VENUS
g day40
DAPI NG2 NKX2.1
h day90
DAPI ASCL1
i day90
DAPI NG2
j P63 mouse colonal
DAPI POMC
k P63 mouse colonal
DAPI NPY
l P63 mouse colonal
DAPI AGRP
m day40
DAPI POMC
n day40
DAPI NPY
o day40
DAPI AGRP
p day5
q day5
DAPI POMC NKX2.1
r day5
DAPI NPY
s day5
DAPI AGRP
t POMC expression
Relative expression levels
hES day40 day90
u NPY expression
Relative expression levels
hES day40 day90
v AGRP expression
Relative expression levels
NS NS
hES day40 day90
w Percentage of POMC-positive areas(%)
day40 day90
x Percentage of NPY-positive areas(%)
day40 day90
y Percentage of AGRP-positive areas(%)
day40 day90

Supplementary Figure 1 | Various properties of Human hypothalamic-pituitary organoids. Related to Figure 1.

(a, b) Immunostaining of a day-90 hypothalamic-pituitary organoid. Two layers of RAX::VENUS (green; a) positive hypothalamic-like tissue in the interior and paired like homeodomain 1 (PITX1) (white; a), LIM Homeobox 3 (LHX3) (red; a), markers of pituitary progenitor cells, and adrenocorticotropic hormone (ACTH) (red; b) positive oral ectoderm-like tissue in the periphery. (c) Culture protocol for hypothalamic-pituitary organoid induction under the condition that BMP4 administration was shortened to three days (d6-d9). (d) Fluorescence microscopy images of hypothalamic-pituitary organoids on day 90 under the condition of shortened BMP4. RAX::VENUS-positive areas (green; d) appear to decrease compared to the original condition. (e) Fluorescence microscopy images of RAX::VENUS (green)-positive cell masses 14 days after isolation from organoids cultured under the shortened BMP4 conditions. RAX::VENUS is expressed only in about half of the cell mass. (f-i) Immunostaining of hESC-derived hypothalamic-pituitary organoids on days 40 (f, g) and 90 (h, i) for ASCL1 (red; f, h), NG2 (red; g, i), co-expressed with RAX::VENUS (green; f) and NKX2.1 (white; g). (j-l) Immunostaining of a coronal section of a nine-week-old mouse brain near the ventral hypothalamus for POMC (red; j), NPY (red; k), and AGRP (red; l). (m-o) Immunostaining of hESC-derived hypothalamic-pituitary organoids on day 40 for POMC (red; m), NPY (red; n), and AGRP (red; o). (p) Phase-contrast microscopy image of a neuron differentiated from hESC-derived hypothalamic-pituitary organoids on day 90. (q-s) Immunostaining of neuronal cells differentiated from hESC-derived hypothalamic-pituitary organoids on day 90. The expression of ventral hypothalamic neuron markers POMC (red; q), NPY (red; r) and AGRP (red; s) were observed. (t-v) Expression of POMC (t), NPY (u) and AGRP (v) on undifferential hESCs, hESC-derived hypothalamic-pituitary organoids on days 40 and 90 (qPCR; n = 3 independent experiments). (w-y) Area of POMC (w), NPY (x), and AGRP (y) expressed in the largest section of the hypothalamic-pituitary organoids using cellSens imaging software (Olympus) (n = 3 independent experiments). Values shown on the graphs represent the means ± S.E.M. *$P<0.05$, **$P<0.01$. Scale bars, 100 μm (a, b, d-s).

Figure2S

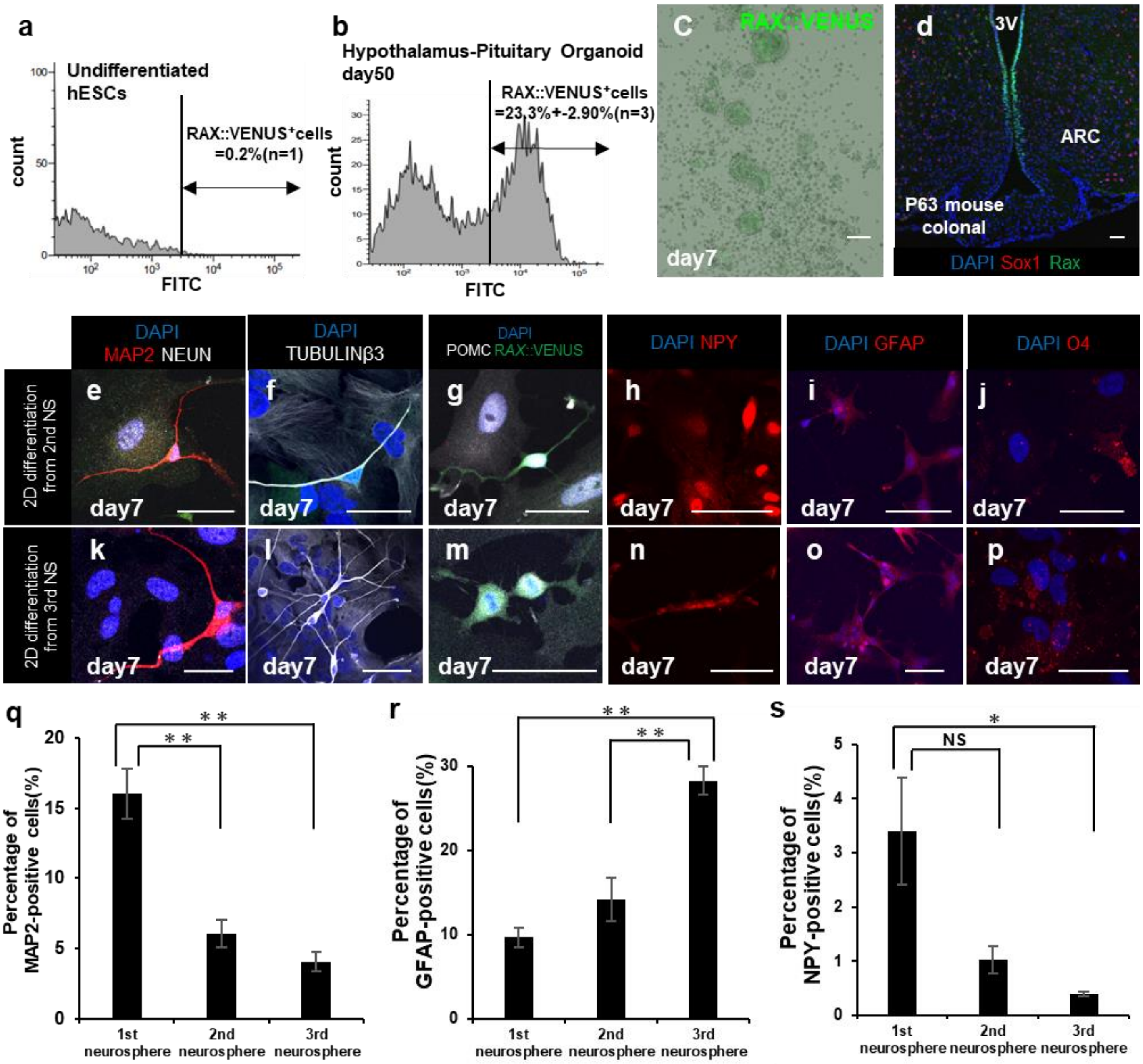
a
Undifferentiated hESCs
RAX::VENUS+cells =0.2%(n=1)
count
FITC
b
Hypothalamus-Pituitary Organoid day50
RAX::VENUS+cells =23.3%+-2.90%(n=3)
count
FITC
C
RAX::VENUS
day7
d
3V
ARC
P63 mouse colonal
DAPI Sox1 Rax
DAPI MAP2 NEUN
DAPI TUBULINβ3
DAPI POMC RAX::VENUS
DAPI NPY
DAPI GFAP
DAPI O4
2D differentiation from 2nd NS
2D differentiation from 3rd NS
e f g h i j
k l m n o p
day7
q
Percentage of MAP2-positive cells(%)
r
Percentage of GFAP-positive cells(%)
s
Percentage of NPY-positive cells(%)
NS
1st neurosphere
2nd neurosphere
3rd neurosphere

Supplementary Figure 2 | *In vitro* characteristics of Sorted RAX::Venus-positive cells Related to Figure 2 and Figure3.

(a) Histogram of fluorescein isothiocyanate (FITC) fluorescence analysis of undifferentiated hESCs as a negative control. (b) Histogram of RAX::VENUS-positive and negative cells in day-50 hypothalamic-pituitary organoids. (c) Fluorescence microscopy images of RAX::VENUS (green)-positive cells isolated from day-90 hypothalamic-pituitary organoids suspended in ultra-low attachment 24-well plates. After seven days, a small number of neurospheres 100-200 μm in diameter were formed, but they did not grow any larger. (d) Immunostaining of a coronal section of a nine-week-old mouse brain near the ventral hypothalamus. Sox1 (red) is not expressed in the Rax (green)-positive tanycytes. (e-p) Immunostaining of neurons and glia differentiated from the neurospheres after multiple passaging. Differentiated cells from the second and third neurospheres expressed the mature neuron markers MAP2 (red; e, k), NEUN (white; e, k), and TUBULINβ3 (white: f, l), the ventral hypothalamic neuron markers POMC (white; g, m) and NPY (red; h, n), an astrocyte marker GFAP (red; i, o), and an oligodendrocyte marker O4 (red; j, p). (q-s) Rates of MAP2-positive cells (q), GFAP-positive cells (r) and NPY-positive cells (s) per each well (total 10^5 cells). Values shown on the graphs represent the means ± S.E.M n=3 independent experiments. *P<0.05, **P<0.01. Scale bars, 50 μm (c-p).

Figure3S

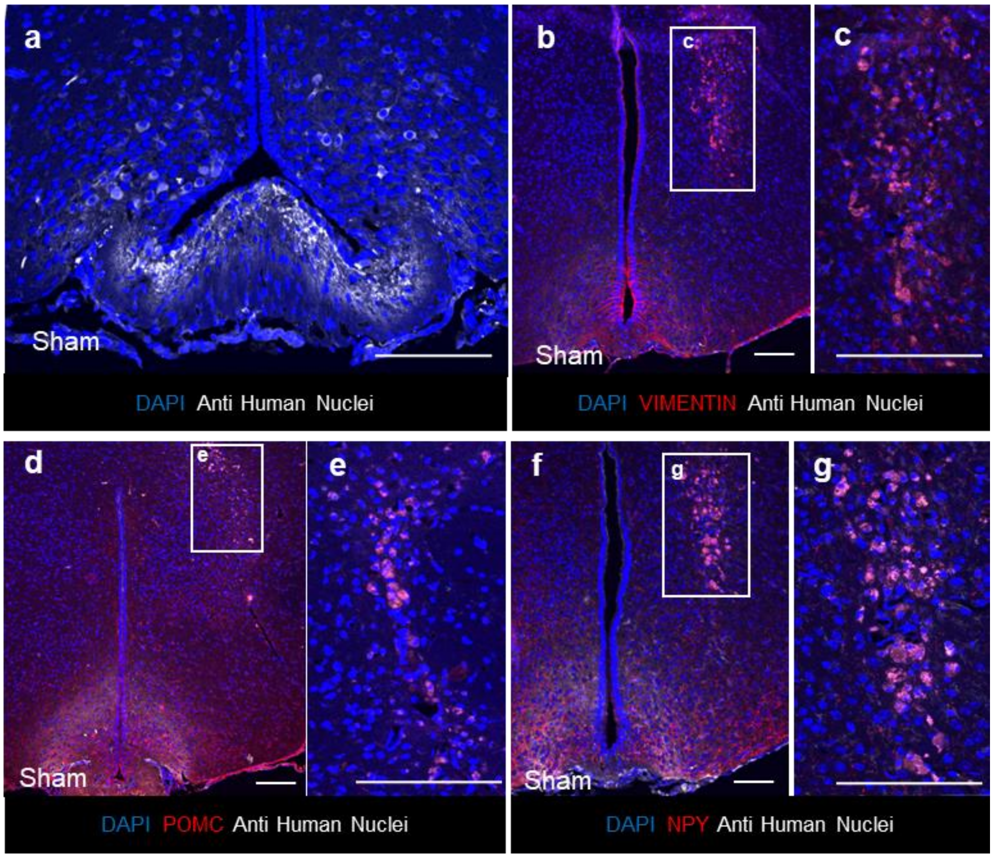

Supplementary Figure 3 | In sham group, anti-human nuclear antibodies and neuropeptides show nonspecific expression and scarring changes. Related to Figure 4.

(a-g) Immunostaining of a coronal section of a nine-week-old sham group mouse brain near the ventral hypothalamus for anti-human nuclear antibodies (white; a-g): VIMENTIN (red; b, c), POMC (red; d, e) and NPY (red; f, g). Scale bars, 100 μm (a-g).

Figure4S

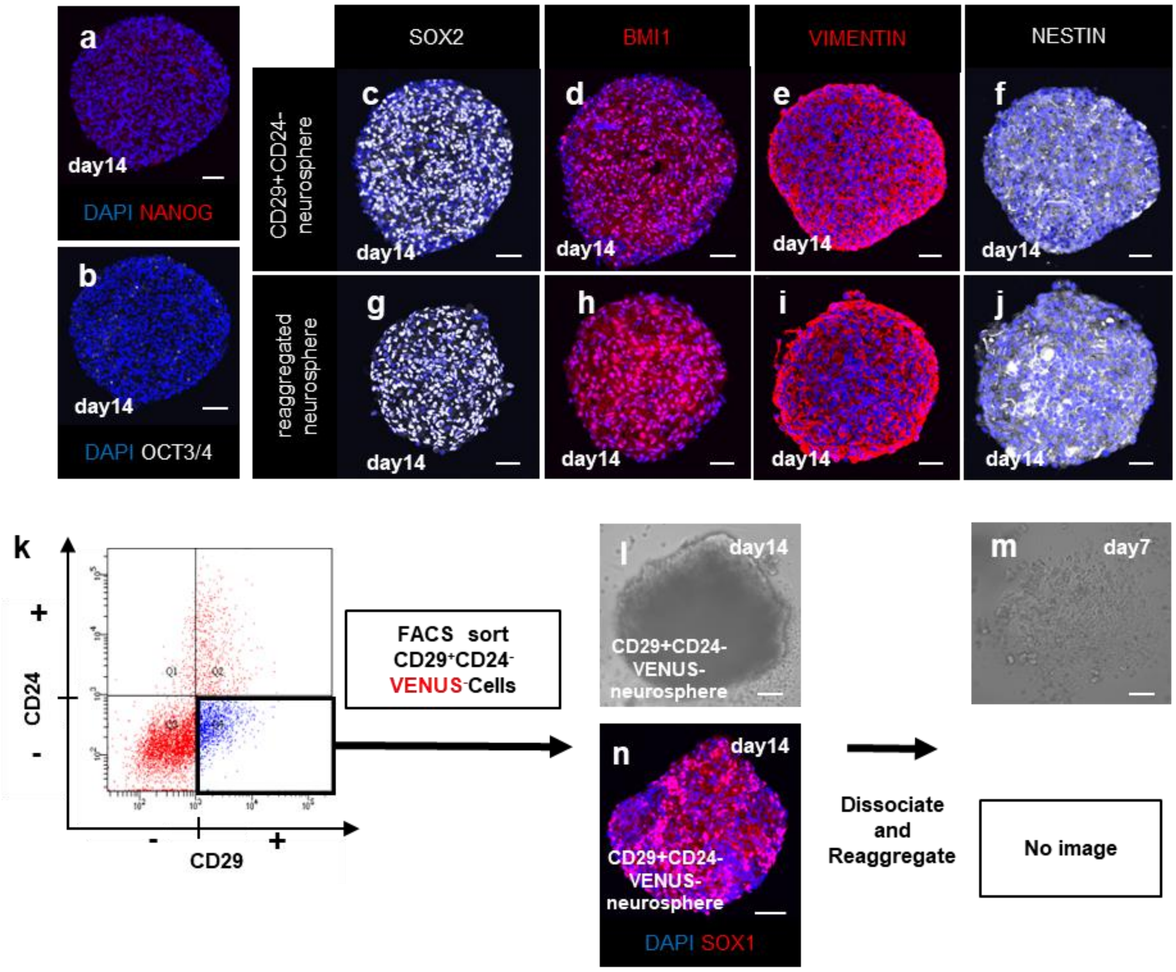

Supplementary Figure 4 | CD29-positive and CD24-negative neurospheres and the reaggregated neurospheres have the properties of neural stem cells but impurities have low aggregation capacity. Related to Figure 5.

(a, b) Immunostaining of the CD29-positive and CD24-negative neurospheres for NANOG (red; a) and OCT3/4 (red; b), undifferentiated human ES cell markers. (c-j) Immunostaining of CD29-positive and CD24-negative neurospheres and reaggregated neurospheres for SOX2 (white; c, g), BMI1 (red; d, h), VIMENTIN (red; e, i), NESTIN (white; f, j), neural stem cell markers. (k) Fluorescence analysis of CD29-positive and CD24-negative cells of human hypothalamic-pituitary organoids. (l) Neurospheres formed by the floating culture of CD29-positive, CD24-negative and RAX::VENUS-negative cells for 14 days (CD29-positive, CD24-negative and RAX::VENUS-negative neurospheres). (m) Microscopy images of cells after one passage from CD29-positive, CD24-negative and RAX::VENUS-negative neurospheres. (n) Immunostaining of CD29-positive, CD24-negative and RAX::VENUS-negative neurospheres for SOX1 (red; n). Scale bars, 50 µm (a-j, l-n).

Figure5S

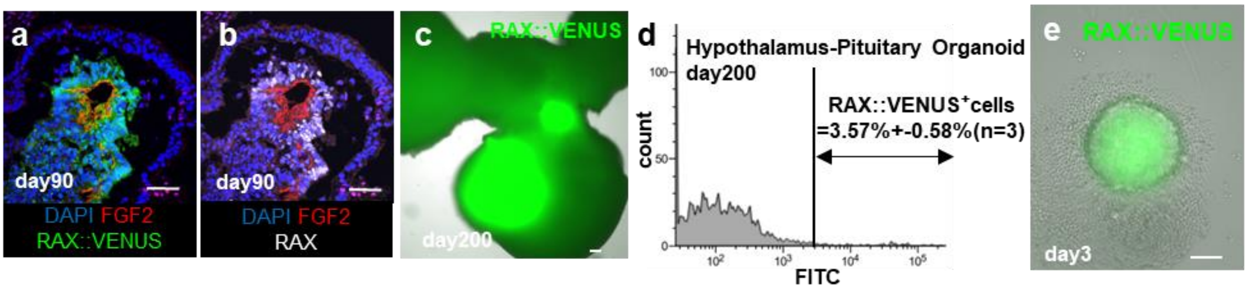

Supplementary Figure 5 | The hypothalamic neural stem cell-like cell niche might exist inside the hypothalamic-pituitary organoids. Related to Discussion.

(a, b) Immunostaining of a day-90 hypothalamic-pituitary organoid. FGF2 (red; a, b) was expressed along the inner cavities of RAX::VENUS (green; a)-positive and RAX (white; b)-positive cells. (c) Fluorescence microscopy images of hypothalamic-pituitary organoids on day 200. RAX::VENUS (green)-positive cells still existed inside the hypothalamic-pituitary organoids. (d) Histogram of RAX::VENUS-positive and negative cells in day-200 hypothalamic-pituitary organoids. (e) Fluorescence microscopy images of the neurospheres composed of RAX::VENUS-positive cells (green) isolated from day-200 hypothalamic-pituitary organoid. Scale bars, 50 µm (a-c, e).

Supplemental Experimental Procedures

Maintenance and differentiation culture of hESCs

The hESCs were used according to the hESC research guidelines of the Japanese government (Nagoya University ES-0001). For the experiments shown, we used the VA22-N37 cell line, which is a RAX::VENUS reporter hESC line established based on KhES-1 (KUIMSe001-A), a biological replicate (Nakano et al., 2012).

Undifferentiated hESCs were maintained on a feeder layer of mouse embryonic fibroblasts inactivated by mitomycin C treatment in DMEM/F-12 (Sigma) supplemented with 20% (vol/vol) KSR (Invitrogen), 2 mM glutamine, 0.1 mM nonessential amino acids (Invitrogen), 5 ng mL^{-1} recombinant human basic FGF (Wako) and 0.1 mM 2-mercaptoethanol under 2% CO_2. For passaging, hESC colonies were detached and recovered *en bloc* from culture dishes by treatment with 0.25% (w/v) trypsin and 1 mg mL^{-1} collagenase IV in phosphate-buffered saline (PBS) containing 20% (v/v) KSR and 1 mM $CaCl_2$ at 37 °C for 10 min. The detached hESC clumps were broken into smaller pieces using a pipette. Passages were performed at a 1:5 split ratio every four days.

For SFEBq culture, hESCs were dissociated into single cells using TrypLE Express (Invitrogen) containing 0.05 mg mL^{-1} DNase I (USA) and 10 μM Y-27632. They quickly aggregated in low-cell-adhesion 96-well plates with V-bottomed conical wells (Sumilon PrimeSurface plate; Sumitomo Bakelite) in differentiation medium (10,000 cells per well, 100 μL) containing 20 μM Y-27632. Differentiation medium (gfCDM) was supplemented with 5% KSR. The gfCDM comprised Iscove's modified Dulbecco medium/Ham's F12 1:1, 1% chemically defined lipid concentrate, monothioglycerol (450 μM), and 5 mg mL^{-1} purified bovine serum albumin (>99% purified by crystallization; Sigma). The SFEBq culture was initiated on day zero. Next, 100 μL of gfCDM per well was added to each well on day 3. From days 6 to 30, the medium was renewed every three days. SAG (Enzo Life Sciences) and recombinant human BMP4 (R&D Systems) were added to the culture medium to reach 2 and 5 nM, respectively, from day 6. BMP4 concentrations were diluted by half-volume changes in BMP4-free medium every third day after day 18. From day 18, the aggregates were cultured under high-O_2 conditions (40%). After culturing in a 96-well plate for 30 days, aggregates were transferred to a 10 cm Petri dish for suspension culture in gfCDM supplemented with 10% KSR and 2 μM SAG on day 30. From day 30, half of the medium was used every three days. The concentration of KSR was increased (final 20% (vol/vol)) from day 50.

Quantitative PCR

Quantitative PCR was performed on ten aggregates per sample. RNAs were purified using the RNeasy kit after treatment with DNase. Quantitative PCR was performed using Power SYBR Green PCR Master Mix and analyzed with the MX3005P system. Data were normalized against the corresponding levels of GAPDH mRNA.

Primers used were as follows:
Forward primer for GAPDH: 5'-GAGTCAACGGATTTGGTCGT-3'
Reverse primer for GAPDH: 5'-TTGATTTTTGGAGGGATCTCCG-3'
Forward primer for POMC: 5'-GAAGATGCCGAGATCGTGCT-3'
Reverse primer for POMC: 5'-ACGTACTTCCGGGGGTTCTC-3'
Forward primer for NPY: 5'-GCTGCGACACTACATCAACCTC-3'
Reverse primer for NPY: 5'-ACACGATGAAATATGGGCTGAA -3'

Forward primer for AGRP: 5'-GGATCTGTTGCAGGAGGCTCAG-3'
Reverse primer for AGRP: 5'-TGAAGAAGCGGCAGTAGCACGT-3'

Immunohistochemistry

Neurospheres, organoids, and mouse brains were fixed in 4% paraformaldehyde (PFA) for 5-20 min. The cells on PDL-coated glass coverslips were fixed with 2% PFA for 10 min, followed by 4% PFA for 15 min. They were immersed in 20% sucrose and embedded in optimal cutting temperature compound (4583; Sakura Finetek, Tokyo, Japan). They were cut into 10 µm thick sections using a cryostat. Immunohistochemistry was performed as described below. The sections (tissue or cells) were washed three times (15 min per wash) in 0.3% Triton X-100/PBS for permeabilization and then washed with PBS three times (15 min per wash). Subsequently, the sections were incubated in 2% (w/v) dry skimmed milk/PBS for 1 h at room temperature (RT) for blocking. The sections were incubated overnight at 4 °C with primary antibodies diluted in 2% dry skimmed milk/PBS. The next day, the sections were washed three times (15 min each wash) with 0.05% Tween 20/PBS and incubated with secondary antibodies diluted in 2% dry skimmed milk/PBS for 2 h at RT. Next, 4,6-diamidino-2-phenylindole (DAPI; D523; Dojindo, Kumamoto, Japan) was added to visualize cell nuclei. Subsequently, the sections were washed three times (15 min each wash) in 0.05% Tween 20/PBS and mounted in Slow Fade™ Diamond (S36972; Thermo Fisher Scientific). ProLong ™ Diamond (P36970; Thermo Fisher Scientific) was used to stain cells on PDL-coated glass coverslips. The primary antibodies were used at the following dilutions:

ACTH (10C-CR1096M1; 1:200; Fitzgerald; RRID: AB_1282437).
AGRP (GT15023; Goat; 1:250; Neuromics; RRID: AB_2687600).
Anti-human nuclei (MAB4383; mouse; 1:1000; Millipore; RRID: AB_827439).
ASCL1 (ab74065; rabbit; 1:200; abcam; RRID: AB_1859937)
BLBP (ab32423; rabbit; 1:100; abcam, Cambridge, UK; RRID: AB_880078).
BMI1 (ab14389; mouse; 1:200; abcam; RRID: AB_2065390).
BrdU (sc-32323; mouse; 1:150; Santa Cruz Biotechnology, Dallas, TX, USA; RRID: AB_626766).
CD24 (ab202073; rabbit; 1:100; abcam; RRID: AB_2904220).
CD29 (integrinβ1) (ab24693; mouse; 1:200; abcam: RRID: AB_448230).
COL23A1 (MA5-24188; mouse; 1:50; Thermo Fisher Scientific; RRID: AB_ 2606945)
COL25A1 (MBS-2561599; rabbit; 1:100; MyBioSource; RRID: AB_ 2927653)
FGF2 (sc-365105; mouse; 1:100; Santa Cruz Biotechnology, Dallas, TX, USA; RRID: AB_10715107).
GFAP (AB5804; rabbit, 1:400; EMD Millipore; RRID: AB_ 2109645).
Ki67 (NCL-Ki67p; rabbit, 1:500; Novocastra; Nussloch, Germany; RRID: AB_442102).
LHX2 (GTX129241; rabbit; 1:200; Genetex, Irvine, CA; RRID: AB_2783558).
LHX3 (AS4002S; rabbit; 1:3000; costom; RRID: AB_2895165),
MAP2 (AB5392; chicken; 1:10,000; Abcam; RRID: AB_2138153).
NANOG (RCAB003P; rabbit; 1:500; Reprocell; RRID: AB_2714012).
NESTIN (PRB315C; rabbit; 1:400; BioLegend, San Diego, CA; RRID: AB_10094393).
NEUN (MAB377; mouse, 1:100; EMD Millipore; RRID: AB_ 2298772).
NG2 (PA5-78556; rabbit; 1:500; Thermo Fisher Scientific; RRID: AB_ 2736211)
NKX2.1 (180221; mouse; 1:200; Zymed (Thermo Fisher Scientific); RRID: AB_86728).

Neuropeptide Y (NPY; 11976; rabbit; 1:3000; Cell Signaling Technology; RRID: AB_2716286).
NR5A1(SF-1) (PP-N1655-0C; mouse; 1:200; Perseus Proteomics; RRID: AB_2904221).
O4 (MAB345; mouse, 1:200; EMD Millipore; RRID: AB_11213138).
OCT3/4(sc-5279; mouse; 1:100; Santacruz; RRID: AB_628051).
PAX6 (PRB-278P; rabbit: 1:250; BioLegend; RRID: AB_ 291612).
PITX1 (MS1568GS; guinea pig; 1:2000; custom; RRID: AB_2895166).
POMC (H02930; rabbit, 1:400; Phoenix Pharmaceuticals, Burlingame, CA; RRID: AB_ 2307442).
RAX (ACG077; guinea pig; 1:10000; custom; RRID: AB_2783560).
Rax (M229; guinea pig; 1:2000 (for tissue 1:500); Takara, Shiga, Japan; RRID: AB_ 2783559).
SOX1 (4194S: rabbit; 1:200; Cell Signaling Technology; RRID: AB_1904140).
SOX2 (GT15098; goat; 1:800 (for tissue 1:200); Neuromics, Edina, MN; RRID: AB_1623028).
TUBULINβ3 (MMS435P; mouse; 1:10,000; BioLegend; RRID: AB_ 2313773).
VIMENTIN (AB5733; chicken; 1:2000 (for tissue 1:1000); EMD Millipore; RRID: AB_11212377).

Cell counting method for SOX1 positive cells

Neurospheres were sectioned at 10 μm intervals, and the section with the longest diameter was selected. Fluorescence immunostaining was performed on the sections, and the number of DAPI-positive cells and SOX1-positive cells were counted visually.

Supplemental Reference

Nakano, T., Ando, S., Takata, N., Kawada, M., Muguruma, K., Sekiguchi, K., Saito, K., Yonemura, S., Eiraku, M., and Sasai, Y. (2012). Self-formation of optic cups and storable stratified neural retina from human ESCs. Cell Stem Cell *10*, 771–785. 10.1016/j.stem.2012.05.009.
